# Highly selective and sensitive recognition of multi-ions in aqueous solution based on polymer-grafted nanoparticle as visual colorimetric sensor

**DOI:** 10.1038/s41598-023-50627-x

**Published:** 2024-01-02

**Authors:** Bahareh Heidari, Pourya Zarshenas, Roya Sedghi, Mohammad Reza Nabid, Rajender S. Varma

**Affiliations:** 1 Department of Polymer and Materials Chemistry, Faculty of Chemistry and Petroleum Sciences, Shahid Beheshti University, GC, Tehran, 1983969411 Iran; 2 Centre of Excellence for Research in Sustainable Chemistry, Department of Chemistry, Federal University of São Carlos, São Carlos, SP 13565-905 Brazil

**Keywords:** Environmental sciences, Chemistry, Materials science, Nanoscience and technology

## Abstract

A novel, selective and sensitive colorimetric sensor for naked-eye detection and adsorption of multi-ions in aqueous solution was synthesized using hybridization of organic–inorganic phase. The polymer-grafted nanoparticles (PGNPs) was synthesized via atom transfer radical polymerization (ATRP) of monomers on modified TiO_2_ NPs and applied under optimized conditions for naked-eye detection: sensor mass: 15 mg; response time: 30 s with limits of detection (LODs) as small as 10, 1, 0.5, and 1 ppb Hg (II), Cd (II), Cu (II), and UO_2_ (II) at pH = 8, 9, 6, and 7, respectively. The efficient selectivity of the naked eye sensor to multi-ions in the presence of various ions was affirmed wherein the color of the chemosensor in the presence of Hg (II), Cd (II), Cu (II), and UO_2_ (II) shifted from gray to violet, orange, green and yellow, respectively. The salient advantages of this method comprise expeditious, selectable, high reproducibility, with reasonable adsorption capacity (133 mg g^-1^) and inexpensive nature for rapid detection of heavy metal ions contamination in aqueous solution in an inexpensive manner. The adsorption mechanism was studied via adsorption kinetics and adsorption isotherm models and the accuracy of the chemosensor has been confirmed and supported by XRD, FT-IR, TGA, ^1^H-NMR, SEM, TEM, EDX mapping, DLS, BET, and EDS analysis.

Environmental contamination of heavy metal ions due to the aftermath of the industrial processes can lead to severe immunotoxic, genotoxic, and neurotoxic effects, especially more toxic heavy metals mainly comprising mercury (Hg), cadmium (Cd), lead (Pb), arsenic (As), and chromium (Cr) species, among others. These pollutants accumulate and concentrate in living organisms and create long-term damaging effects that lead to severe ecological and severe health problems^1^. According to the recommendation of the World Health Organization, the standard water quality limit is ~ 10 ppb Hg, Cd, Pb, and related toxic metal ions^2^ and consequently, several methods have been widely deployed for the detection and measurement of toxic metal ions in aqueous solution. The most common techniques followed are atomic spectrometry^3,4^, voltametric methods^5^, and molecular spectrophotometry^6^. Since the monitoring of the mercury levels in the environment is essential, the common suggested techniques for determination of mercury ions include the inductively coupled plasma mass spectrometry (ICP-MS)^7^, and inductively coupled plasma atomic emission spectrometry (ICP-AES)^8,9^ as they are devoid of any preconcentration step. Although these methods have better diagnostic limits for determining a wide linear range but these instruments are high-cost analysis tools. Additionally, the transport and collection of the necessary amount of sample is a source of numerous possible hinderances including the issue of sampling expertise as well as human error. However, smaller, portable and inexpensive devices have been marketed in recent years^10–14^. Among these, the optical sensors have garnered immense attention as they have many appliances in a wide variety of areas like chemistry, biology, and environmental monitoring^15^. The use of chemical sensors is one of the most advanced methods in chemistry that enables the quantitative measurement of various species instantaneously. Chemical optical sensors are among the youngest chemical sensors that enable online and field surveillance. Ideally, a sensor ought to be of sufficient sensitivity and high selectivity for the target species, fast response time, good signal-to-noise ratio, besides simplicity, reliability, and economical aspects. Existing sensors have limitations in terms of insufficient long-term stability, interference from other species, and inadequate detection limits. However, the underlying motivation and the studies on optical sensors are increasing. The colorimetric sensors possess the advantages of simple and low-cost preparation, high sensitivity, reasonable selectivity, and no requirement for separate reference apparatus. Dithizone (diphenylthiocarbazone, DTZ) is known to as a selective and sensitive chromogen with effective chelating agents for the visual detection of heavy metal ions; Refs.^16,17^ immobilization of the ligand being a critical step in the fabrication of visual chemosensors^18^. The immobilization of indicator chromogen has been carried out using physical^19,20^ or chemical entrapment on the support matrixes^21^ with both the strategies have several advantages and disadvantages. Physical immobilization is an easy process, but the colorimetric chemical sensors will have relatively short stability due to the leaching of ligand into solution. Thus, chemical immobilization via covalent binding of ligand onto the support matrixes is the superior technique for preparing the chemical sensors with higher stability and reproducible response.

The main problem of the DTZ ligand is its insolubility in aqueous medium. The design and synthesis of PGNPs via ATRP process deploying hydrophilic monomers offer the high dispersion of nanocomposites^22^. Acrylamide (AM) has been chosen as the highly hydrophilic monomer to modulate the surface properties of a hydrophobic material such as DTZ chromogen. The concentration of toxic heavy metal ions is usually below the detection limits (LOD), so the preconcentration of these ions is necessary before the measurement wherein three-dimensional and hydrophilic structures can be used for this purpose. *N,N*-methylenbisacrylamide (MBA) is good candidate as a cross-linker for the adsorption of toxic metal ions from an aqueous solution. The salient feature of using AM and MBA in this study is that the nitrogen and oxygen heteroatoms in their structure renders the ensuing PGNPs as an ideal structure for interaction with ions. Thus, the combination of better sensing and superior adsorbent of toxic metal ions has been developed for the effective detection, removal, and recovery of ions in aqueous media; specific surface area and phase composition of TiO_2_ NPs are efficient agents in this application. The hydrophilic functionalization of PGNPs can effectively disperse the final nanocomposites in aqueous solution. The PGNPs was synthesized via a surface-initiated, atom-transfer radical polymerization of AM, MBA, and *f*-DTZ on modified TiO_2_ NPs. The synthesis and application of chemosensor is schematically is depicted in Fig. 1.

**Figure 1:**
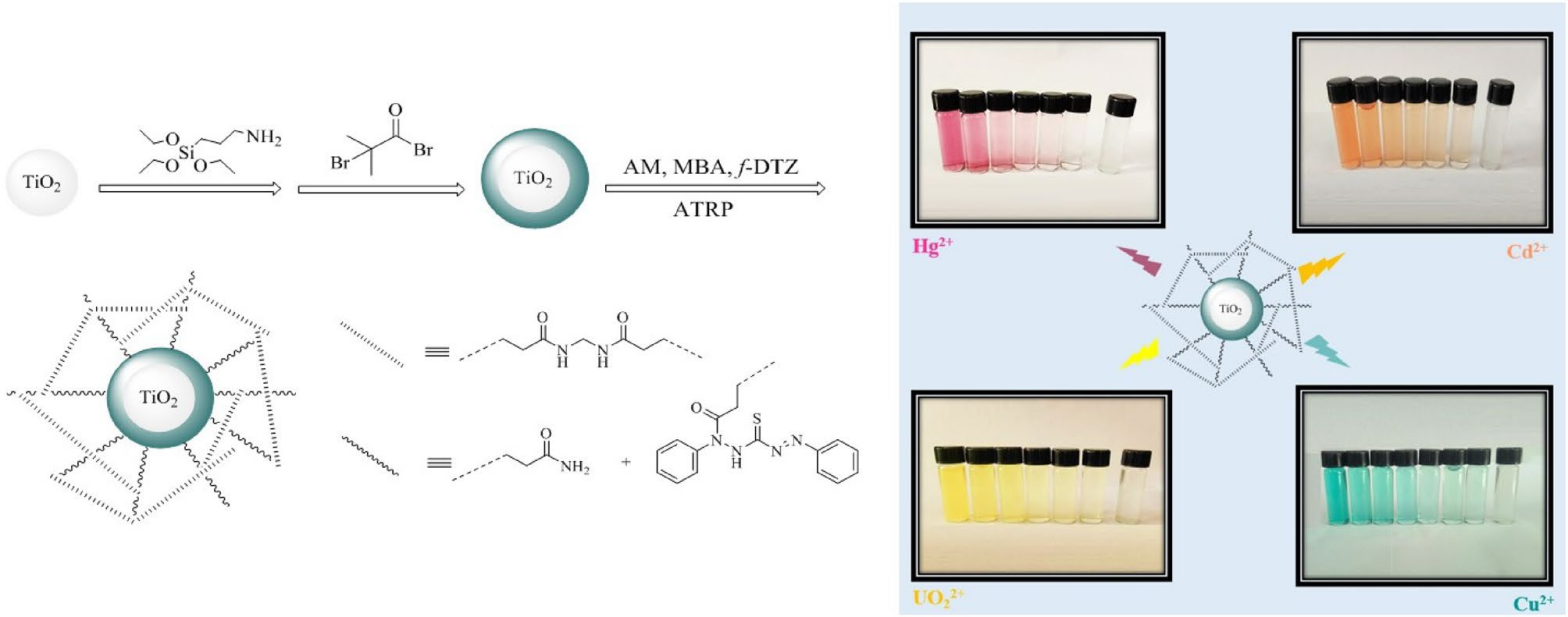
The synthesis and application of chemosensor.

## Experimental

### Reagents and materials

Ammonium(II) sulfate, Titanium (IV) chloride, Ammonium hydroxide, Acrylamide (AM), Acrylic Acid (AA), Dithizone (DTZ), *N,N,N',N'',N''*- Pentamethyldiethylenetriamine (PMDETA, ≥ 98%), 4-Dimethylaminopyridine (DMAP), *N,N*′-Dicyclohexylcarbodiimide (DCC), (3-Aminopropyl)triethoxysilane (APTES ≥ 98%), 2-Bromoisobutyro bromide (BiBB 98%), Copper(I) chloride (CuCl), Copper(II) chloride (CuCl_2_), Copper(II) nitrate, Cadmium acetate, Magnesium sulfate, Calcium sulfate, Potassium chloride, Sodium chloride, N,N-Dimethylformamide (DMF), Tetrahydrofuran (THF), Dimethyl sulfoxide (DMSO), Isopropyl alcohol, Toluene, and Triethylamine (TEA) were all the reagents of analytical grade purchased from Merck and Sigma-Aldrich Companies, and double distilled water was used in all the experiments.

### Preparation of TiO_2_ NPs

The preparation of the TiO_2_ NPs anatase phase was carried out in the following way. First, 9 g of ammonium sulfate was dissolved in 50 mL of distilled water, and 4 mL TiCl_4_ was slowly added to the mixture reaction. After the addition was completed, the reaction was heated at 75 °C for 90 min. The washed product was dried in an oven, and the obtained precipitate was calcined at 350 °C for 4 h.

### Preparation of amine-functionalized TiO_2_ NPs

Silane derivative-based functionalization strategies offer several advantages, namely the improved structural stability against aggregation and the creation specific functional groups. In this regard, NPs surface modification with APTES was carried out as follows: 1 g TiO_2_ NPs was dispersed in toluene (20 mL) by sonication. Then, 2 mL APTES was added to this mixture reaction, and the solution was subjected to mechanical stirring at room temperature for 24 h. The final product was collected by centrifugation and washed with ethanol/distilled water and dried at room temperature. The formation of a layer of amino silane on TiO_2_ NPs facilitates the linking of the required Br agent for the ATRP process.

### Synthesis of TiO_2_-Br

The TiO_2_-APTES (1g) was taken in the three-necked flask loaded with THF (30 ml) and TEA (2.25 mL). The solution was vigorously stirred for 30 min at 0 °C. After purging with the N_2_ atmosphere, 2.25 mL of 2-bromoisobutyryl bromide was added drop by drop, with continuous stirring at 0 °C for 1.5 h and followed by stirring for 24 h at room temperature. The TiO_2_-Br NPs were separated by filtration followed by washing several times with THF and ethanol to attain purity and finally, dried under vacuum at 60 °C for 24 h.

### Synthesis of functionalized Dithizone (f-DTZ)

To synthesize functionalized DTZ, 50 µL acrylic acid was mixed with 0.1 g of DMAP and 0.24 g of DCC dissolved in 15 mL of DMSO under vigorous stirring at °C for 2 h. Then 0.3 g DTZ was added to the solution and stirred at 60 °C for 24 h. Finally, the product was filtrated and washed with DMSO/distilled water, and dried for 24 h at 50 °C.

### Grafting the AM, MBA and f-DTZ onto the TiO_2_ NPs (poly(AM-MBA-DTZ)-grafted TiO_2_ NPs)

For the polymerization of monomers on the surface of modified NPs, AM (0.5 g), MBA (0.3 g), and *f*-DTZ (0.1 g) were dissolved in DMF/H_2_O (3 mL) with a 2.5/1 ratio and 0.15 mL isopropyl alcohol. Then 0.02 g of CuCl and PMDETA (0.4 mL) were added to the reaction mixture under the N_2_ atmosphere, and followed by the addition of 0.002 g of CuCl_2_. The solution was kept under a bubbling atmosphere (N_2_) for 15 min, then the TiO_2_-Br NPs (0.15 g) were dispersed in 0.25 mL of H_2_O using an ultrasonic bath and added to reaction mixture, and the polymerization process was carried out at 75 °C for 48 h. The ensuing product was filtered and washed with DMF, ethanol, and distilled water. Finally, it was dried in a vacuum oven overnight at 35 °C.

### Instruments

Infrared spectra analyses of the products were recorded using KBr via a BOMEM MB-series spectrometer between the frequency ranges of 4000 and 400 cm^−1^. Flame atomic absorption (FAAS) measurements were recorded on a Shimadzu model AA-6650. Standard hollow-cathode lamps were used as a light source for Cu and Cd determination. The pH measurements of the metal ions solutions were carried out by Metrohm 827 equipped with a combined glass calomel electrode. A Shimadzu model 2100 UV–vis spectrophotometer with a 10 mm quartz cell was deployed for the determination of optical absorption spectra of the colorimetric sensor. The morphologies, particle sizes, and distribution of the pristine TiO_2_ NPs and PGNPs were evaluated using scanning electron microscopy (SEM) (Philips XL-30) and transmission electron microscopy (TEM) (Philips CM‐30). The thermal decomposition and investigated stability of the PGNPs were applied using thermogravimetric analysis (TGA) with a flow rate of 10 °C/min^−1^ and the heating range from 25 to 800 °C. The crystalline phases were identified by X-ray diffraction (XRD) patterns with Siemens D5000 diffractometer with monochromatised CuKα radiation of $\lambda$=1.54060 A at a scanning rate of 10 ^◦^/min over the range of 2θ = 10–80° at room temperature. The elemental analysis of nanocomposite was investigated using energy dispersive X-Ray spectroscopy (EDX).

## Results and discussion

### Characterization of the chemosensor

#### The XRD analysis

**Figure 2:**
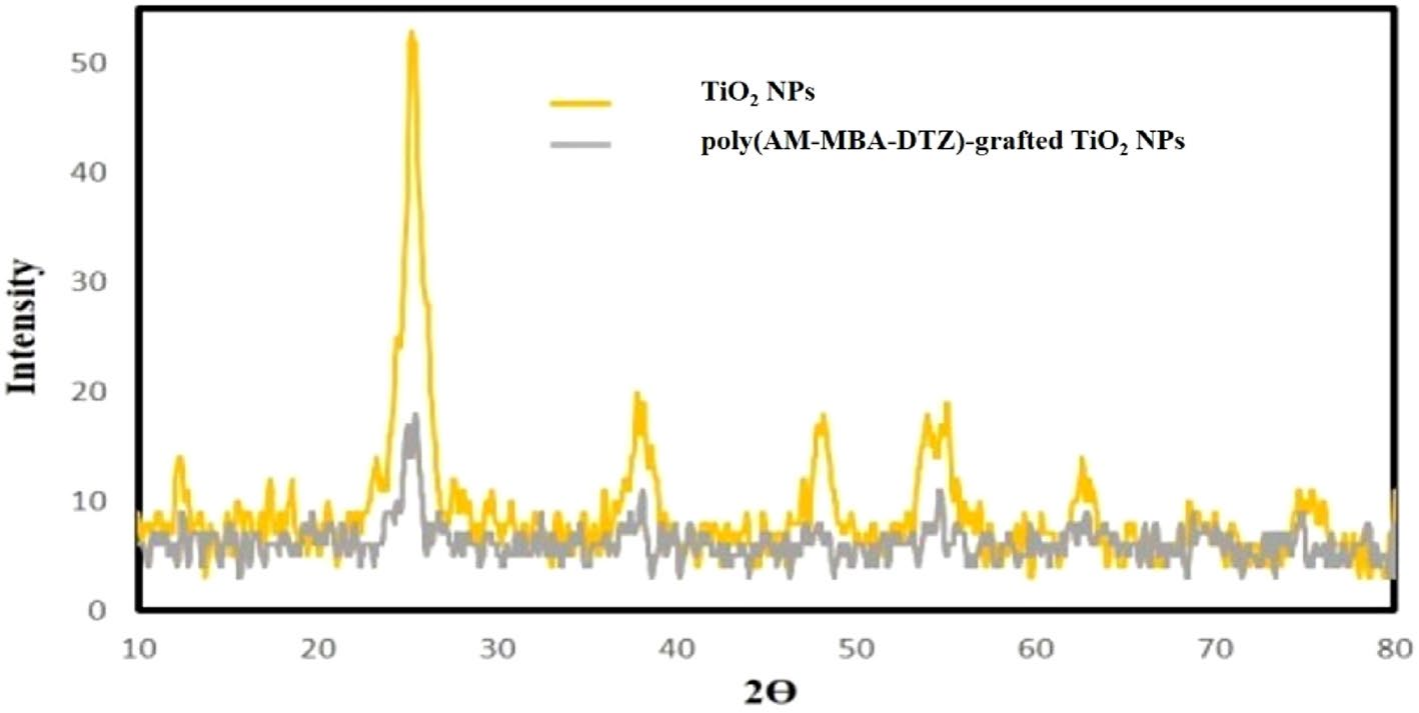
XRD pattern of TiO_2_ NPs and poly(AM-MBA-DTZ)-grafted TiO_2_ NPs.

X-ray diffraction technique is typically used to investigate and identify phase formation and obtain information about the crystallinity of the TiO_2_ NPs and poly(AM-MBA-DTZ)-grafted TiO_2_ NPs. The obtained X-ray diffraction patterns for TiO_2_ NPs and polymeric matrices are shown in Fig. 2. The diffraction patterns and peak positions of TiO_2_ NPs well agreement with those of tetragonal TiO_2_ anatase phase (2θ = 25.35, 37.77, 47.83, 54.22, 62.92, 75.40)^23,24^. No peak corresponding to any secondary and, or impurity phases was visible, indicating the good purity of the NPs. The amorphous nature of the polymer matrices can be observed by reduce intensity of peaks and the appearance of a broad peak at 2ϴ = 10–30.

### IR spectra

**Figure 3:**
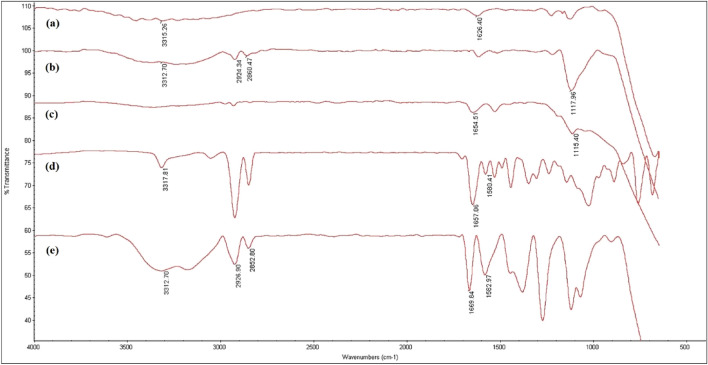
FT-IR spectra of TiO_2_ NPs (**a**), TiO_2_-APTES (**b**), TiO_2_-Br (**c**), *f*-DTZ (**d**), poly (AM-MBA-DTZ)-grafted TiO_2_ NPs (**e**).

The FT-IR spectrum of TiO_2_ NPs exhibited broadband in the low-frequency region (750–500 cm^−1^) that can be assigned to vibrations of Ti–O–Ti^25^. The band at 1626 cm^−1^ and 3200–3300 cm^−1^ were indexed to the stretching vibration of adsorbed water on TiO_2_ NPs surface and the presence of hydroxyl groups of NPs surface, respectively (Fig. 3a). The spectra of functionalized TiO_2_ NPs using APTES, a peak at 1117 cm^−1^, and two bands in the region of 2860–2924 cm^−1^ were ascribed to Ti–O–Si groups^26^ and C–H stretching vibrations of the anchored propyl group^27^, respectively, which confirmed the coverage of silane agent with a free amine group on the TiO_2_ NPs surface (Fig. 3b). In the spectrum of TiO_2_-BiBB, peaks corresponding to –NH–CO– vibrations at 1654 cm^-1^ was indicative of the successful attachment of ATRP initiator functional moiety onto the surface of TiO_2_-APTES (Fig. 3c)^28^. In the spectrum of f-DTZ, the appearance of the band at 1657 cm^−1^ was accredited to the amide group due to the successful converge of DTZ to AA (Fig. 3d). The spectrum of the poly(AM-MBA-DTZ)-grafted TiO_2_ NPs indicated all bands related to TiO_2_ NPs, AM, MBA and *f*-DTZ. The strong peak at 1669 cm^−1^ according to –NH–CO– vibrations in amide monomers, which indicated the successful polymerization of monomers on the surface of TiO_2_-Br NPs (Fig. 3e).

### EDX mapping

**Figure 4:**
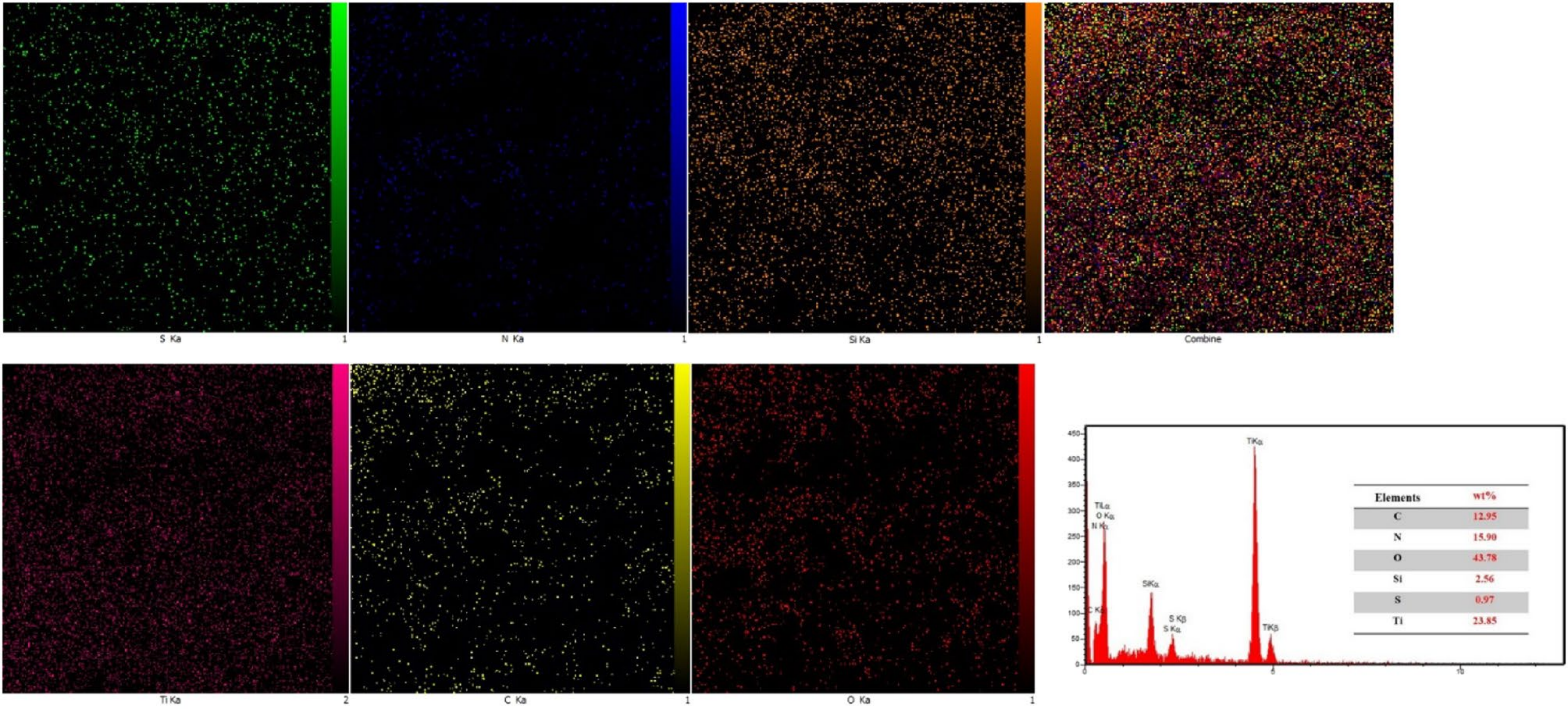
MAP pattern and corresponding EDX spectrum of poly(AM-MBA-DTZ)-grafted TiO_2_ NPs.

The chemical elemental composition and distribution of the poly(AM-MBA-DTZ)-grafted TiO_2_ NPs were further investigated via EDX spectrum and energy dispersive X-ray mapping (Fig. 4). EDX measurements verified the presence of S, N, Si, Ti, C and O elements in the product, which are in good agreement with the results of other characterizations. Further, the chemical distribution of the PGNPs was studied using EDX elemental mapping. The representative corresponding EDX mappings of S, N, Si, Ti, C and O elements illustrated extensive dispersion of elements on the PGNPs surface.

### SEM

**Figure 5:**
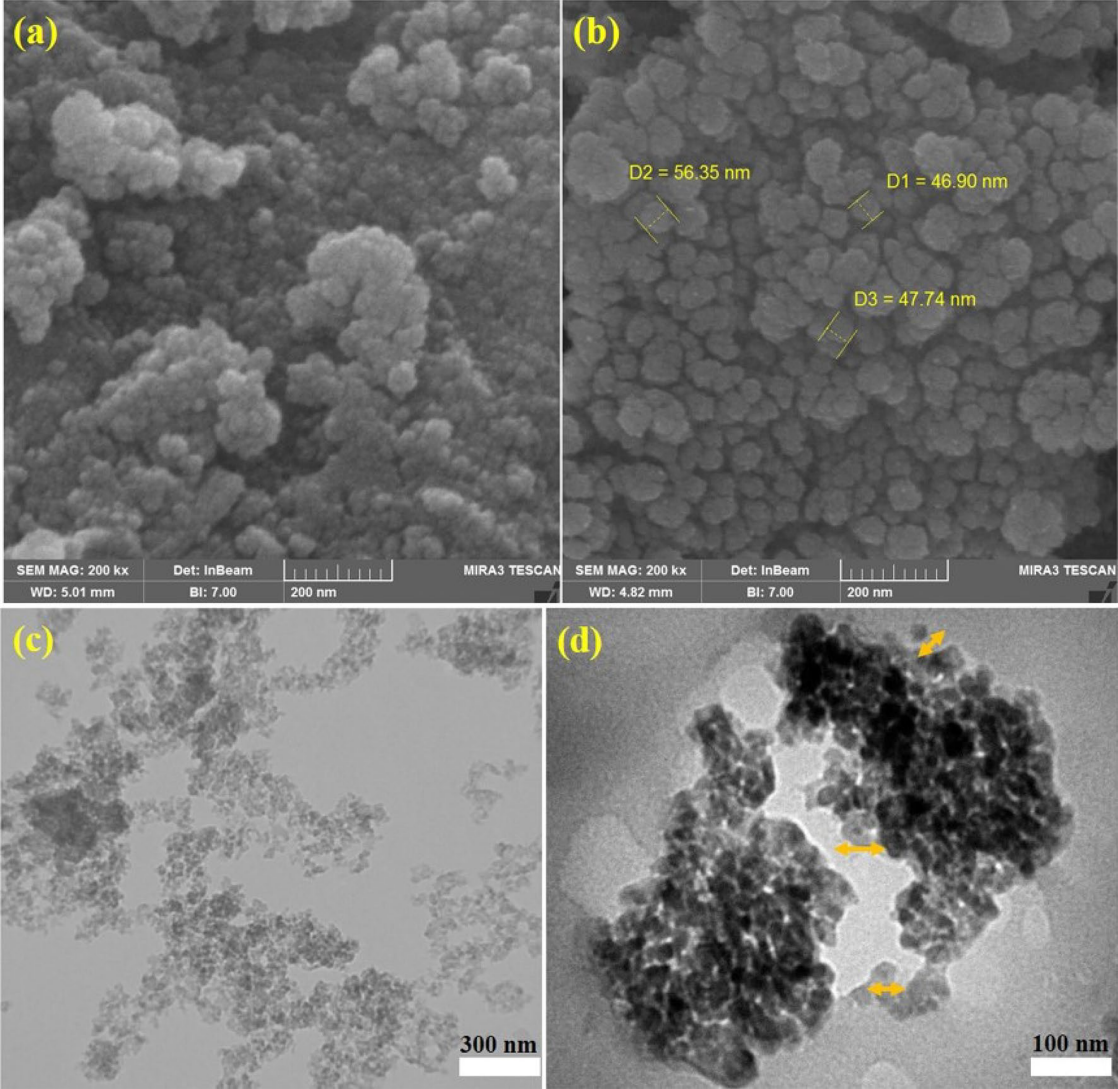
SEM imaging of TiO_2_ NPs (**a**), poly(AM-MBA-DTZ)-grafted TiO_2_ NPs (**b**) and TEM imaging of TiO_2_ NPs (**c**) and poly(AM-MBA-DTZ)-grafted TiO_2_ NPs (**d**).

Figure 5 shows SEM images of pure TiO_2_ NPs and poly(AM-MBA-DTZ)-grafted TiO_2_ NPs. Figure 5a validated that highly ordered nano-spherical shapes were formed; average pore diameter of these nano spherical shape as calculated from SEM images is 15–25 nm. In Fig. 5b, the observation of particles size distribution in the range of 40–60 nm, confirmed the formation of a polymeric coating on the surface of NPs.

### TEM

The morphology of the pure TiO_2_ NPs and poly(AM-MBA-DTZ)-grafted TiO_2_ NPs were investigated, and is shown in Fig. 5c and d. The TEM images reveal that the TiO_2_ NPs consist of well-dispersed spherical structures with a diameter varying from 15 to 25 that is in agreement with SEM result and confirm that the polymeric layer uniformly attached to the TiO_2_ NPs surface.

### TGA analysis

**Figure 6:**
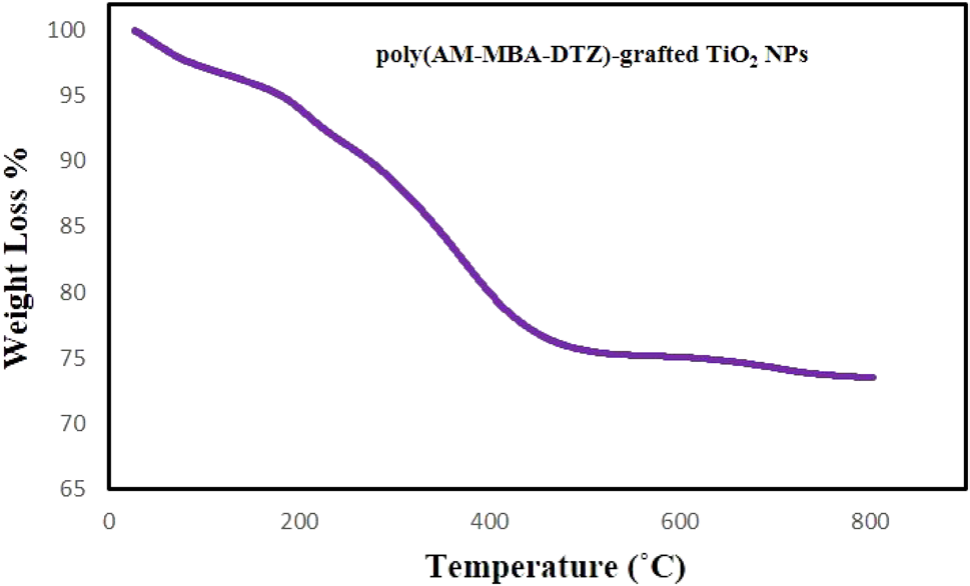
TGA analysis of poly(AM-MBA-DTZ)-grafted TiO_2_ NPs.

Figure 6 shows the thermal analysis of poly(AM-MBA-DTZ)-grafted TiO_2_ NPs at high temperatures ranging from 25 to 800 °C. The initial weight losses from room temperature to about 200 ˚C is attributed the removal of surface moisture content. The significant weight loss of the PGNPs was observed in the second stage when the temperature is increased from 200 °C to 750 °C which can be attributed to the degradation of polymer (AM-MBA-DTZ) on the surface of NPs. A total of 26.54% weight loss occurred in the PGNPs structure.

### NMR spectroscopy

**Figure 7:**
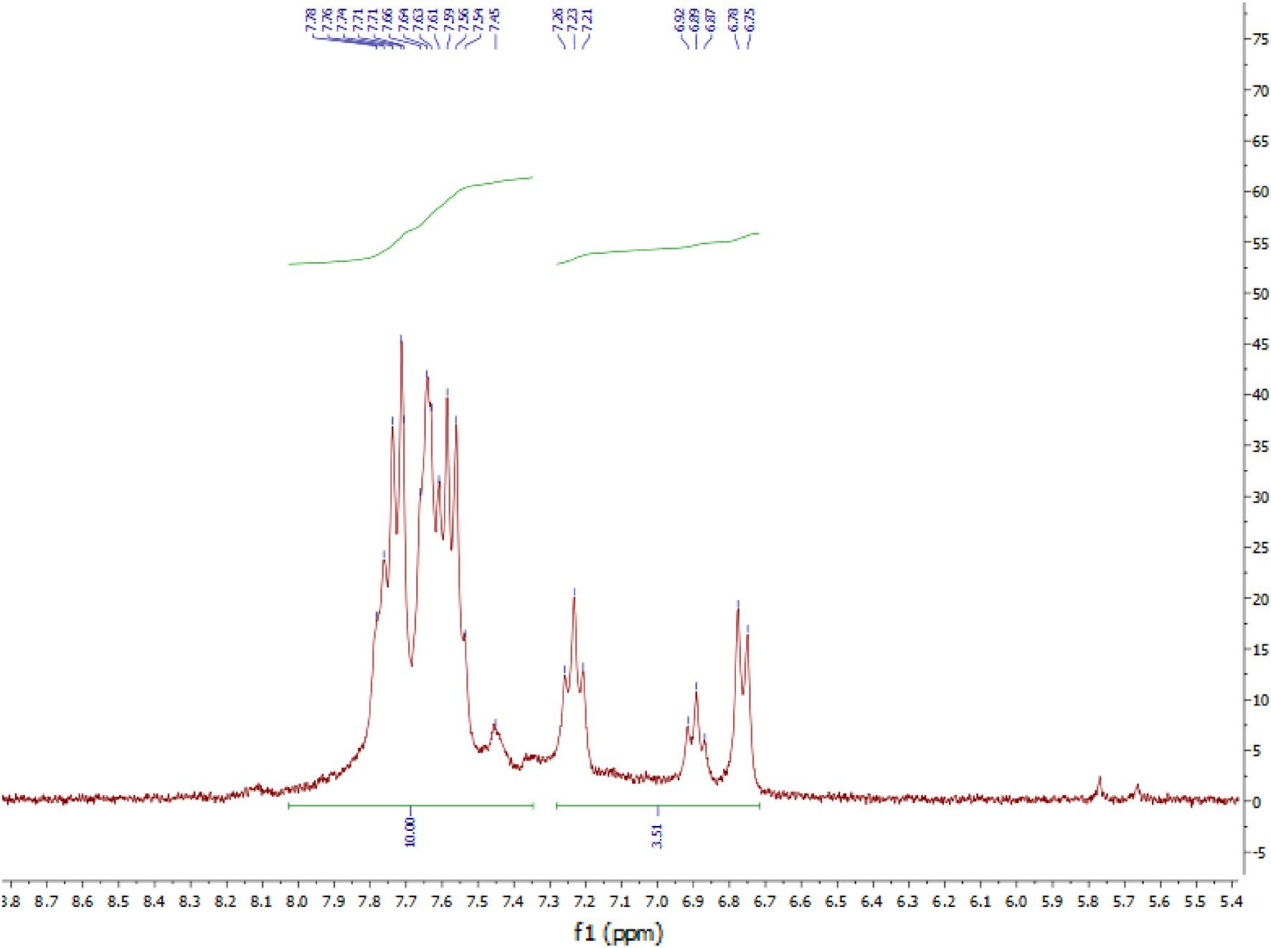
The ^1^H-NMR spectra of *f*-DTZ.

The NMR analysis of the modified DTZ illustrates peaks at 6.70–7.25 ppm and 7.40–7.90 ppm with the integral of ~ 3 and 10, which are related to the acrylate group and aromatic hydrogens, respectively. Comparing the acrylate hydrogens (3H appeared at 6.70–7.25 ppm) with the aromatic hydrogens (10 H appeared at 7.40–7.90 ppm) confirms the successful modification of DTZ (Fig. 7).

### Dynamic light scattering (DLS)

**Figure 8:**
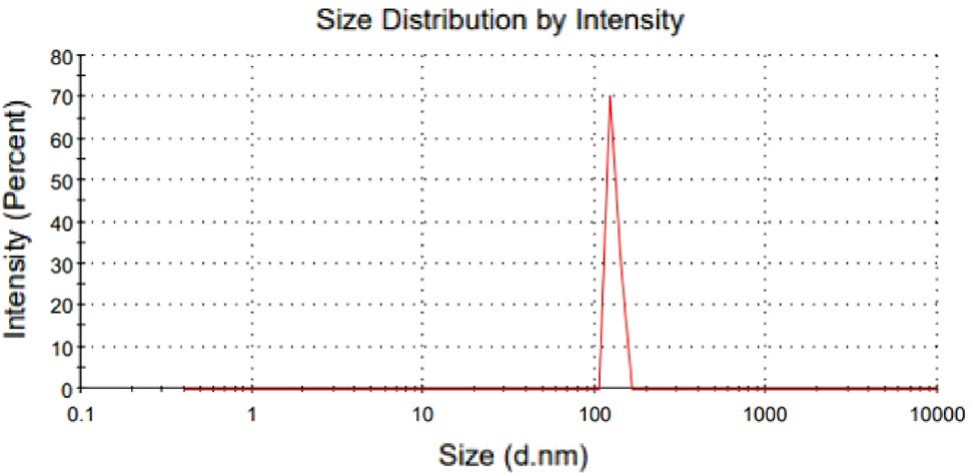
DLS data, hydrodynamic diameter of poly(AM-MBA-DTZ)-grafted TiO_2_ NPs.

The DLS of poly(AM-MBA-DTZ)-grafted TiO_2_ NPs was measured and the hydrodynamic diameter 204/5 (d.nm) was obtained (Fig. 8). The differences between the conditions required by the two measurement techniques were the reason for the observed discrepancy in the diameter of the particles detected by TEM and DLS. The hydrodynamic diameter of the particles, including the core and any molecules connected to the surface such ions and solvent/water molecules, is estimated using DLS analysis since it necessitates an NPs suspension. In contrast, TEM visualizes the dehydrated NPs without any associated molecules^29^. On the other hand, as the structural features of the synthezied PGNPs, and the presence of hydrophilic membrane in the structure, the water adsorption, hydrogen bonding of amide groups with water, swelling, and thus an increase in hydrodynamic diameter were predictable.

### BET analysis

**Figure 9:**
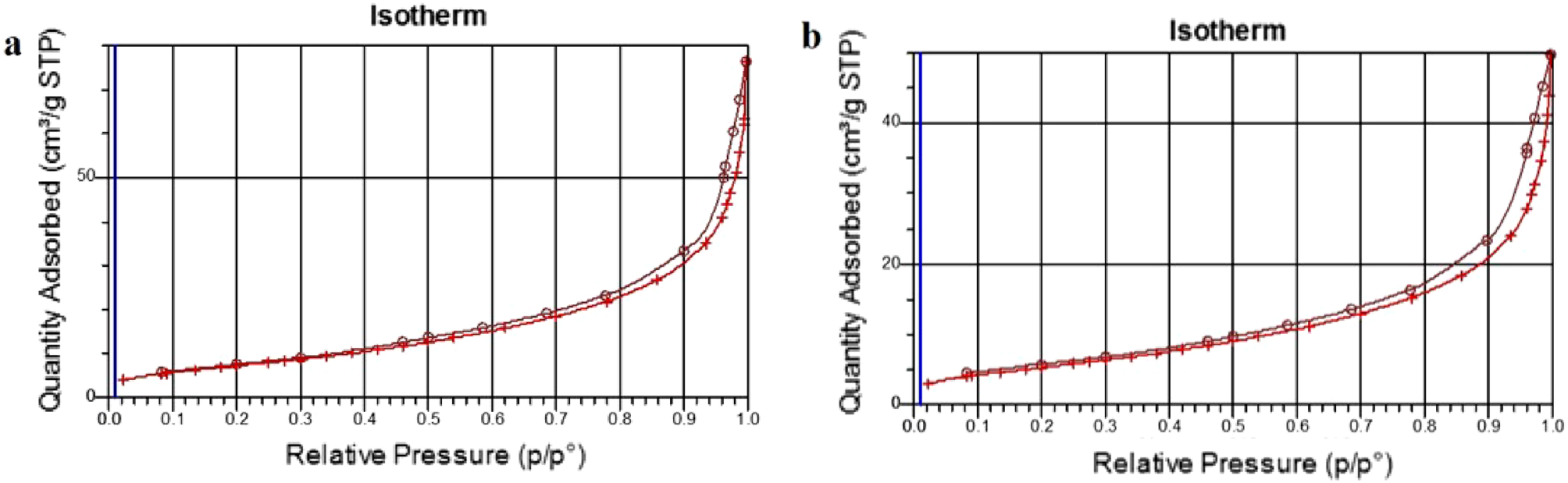
N_2_ adsorption–desorption isotherm of poly(AM-MBA-DTZ)-grafted TiO_2_ NPs (**a**) and poly(AM-MBA-DTZ)-grafted TiO_2_ NPs@Cd(II) (**b**).

The BET surface area of the poly(AM-MBA-DTZ)-grafted TiO_2_ NPs and poly(AM-MBA-DTZ)-grafted TiO_2_ NPs@Cd(II) were found to be 27.45 m^2^/g and 19.96 m^2^/g, respectively. The results displayed that the BET value of chemosensor after adsorption of Cd ions was reduced. The surface area of the used chemosensor decreased, probably due to sorption of Cd ions. Figure 9a,b shows the N_2_ adsorption–desorption isotherm of PGNPs without and with metal ion loading, respectively.

### Colorimetric and optical studies

Colorimetric studies were performed to investigate the naked-eye sensing behavior of the synthesized chemosensor at various target ions concentrations. Compared with the other methods, the application of the colorimetric sensors was simple, fast, without any need for bulky devices, and can be performed without any specialized skilled manpower. For the naked-eye sensing, specific solutions of Hg, Cd, Cu, and UO_2_ ions (various concentrations) were prepared in distilled water. Colorimetric sensing effect of diverse amount synthesized sensor (5–25 mg) in varying concentration ranges (0.5 ppb−1000 ppm) of mentioned ions in the range of pH = 2–10 and time duration of 1–120 s, were also examined. To achieve ideal sensor performances for the naked-eye and optical detection of ions, pH- responses and time experiments were optimized.

### Parameter optimization of the colorimetric sensor

#### pH- responses

**Figure 10:**
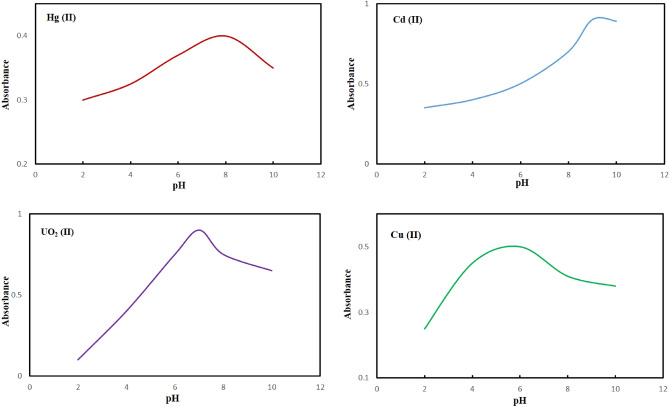
The effect of pH on the metal sorption capacity and detection for Hg (II), Cd (II), Cu (II) and UO_2_ (II). Concentration of ions: 1 ppm, colorimetric sensor amount: 15 mg at room temperature for 2 min.

The effect of pH was investigated in terms of enhanced efficiency. Briefly, the pH of solutions was adjusted in the range of 2–10, and then each of the samples was mixed with 15 mg of sensor and stirred at room temperature for 2 min. The clear color changes were detected via naked-eye and optical measurement was carried out through UV–vis spectra. All the experiments were repeated three times to achieve high accuracy results. Figure 10 depicts an enhanced efficiency adsorption capacity to pH = 8.0, pH = 9, pH = 6, and pH = 7 for Hg (II), Cd (II), Cu (II), and UO_2_ (II), respectively wherein the mentioned pH’s are chosen for the following experiments. The diminished response at acidic pH in all experiments be justified due to the protonation of heteroatoms of PGNPs from the aqueous solution and, therefore, its capability towards extraction of toxic ions into the polymeric membrane phase is extremely reduced.

### Optimization of time

To assess the effect of detection time, the naked-eye and optical detection processes were investigated. The response time of the chemosensor is estimated by the time required for the measuring ions to diffuse from the solution toward PGNPs to associate with the ligand. The resulted response time of the chemosensor depends on several factors such as the pH of the sample solution, the concentration of the measuring ions, and well dispersity of the chemosensor. The time-dependent response characteristics of the optical chemosensor were observed wherein the absorbance increased remarkably with the increase of reaction time, and the response of the chemosensor was found to reach ~ 99% in 30 s; when the time was longer than 30 s, the color change was hardly ever demonstrated. Thus, 30 s could be used as the optimal detection time for ions. Therefore, the naked eye detection of Hg (II), Cd (II), Cu (II), and UO_2_ (II) by colorimetric sensor could be carried out in pH = 8.0, pH = 9, pH = 6, and pH = 7 with the interaction time of 30 s.

### Naked-eye response

**Figure 11:**
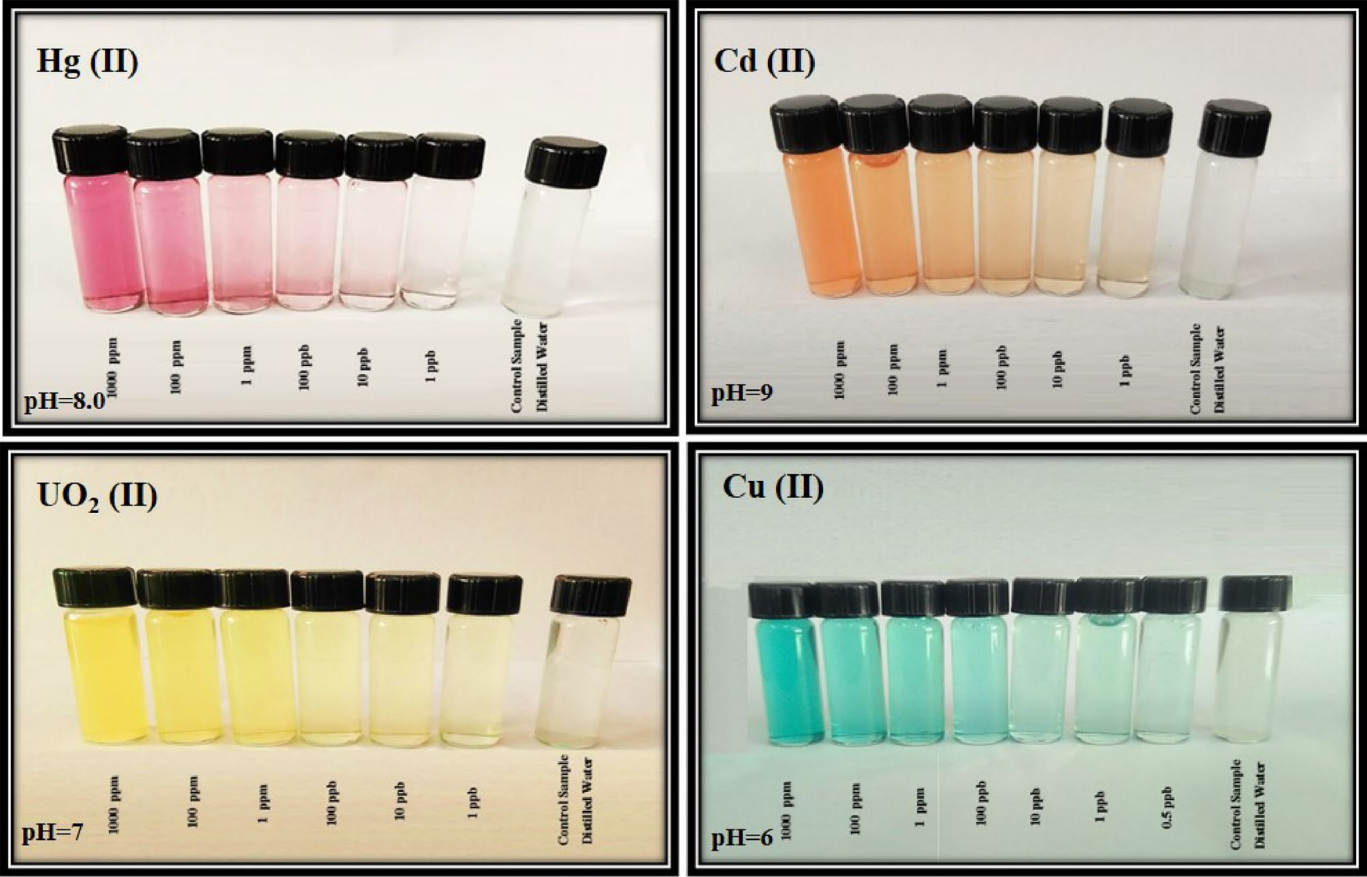
Colorimetric sensor response for Hg (II) at pH = 8.0, Cd (II) at pH = 9.0, Cu (II) at pH = 6.0 and UO_2_ (II) at pH = 7.0 in 30 s.

The Naked eye response of the proposed multi-ions selective sensor at varying ions concentrations under optimal experimental conditions, is shown in Fig. 11. The interaction between ions and selective sensor lead to color change from gray to violet, orange, green and yellow for Hg, Cd, Cu, and UO_2_ ions, respectively, and color change increase with increasing concentration of ions. The clear naked eye detection was observed over a wide range [(0.5 ppb–1000 ppm)] with a low detection limit of 10 ppb, 1 ppb, 0.5 ppb and 1 ppb for Hg (II), Cd (II), Cu (II), and UO_2_ (II) at pH = 8.0, pH = 9, pH = 6, and pH = 7, in 30 s, respectively. To the best of our knowledge, combining nano surface structure, and hydrophilic membrane provide not only a high dispersity of hydrophobic indicator in aqueous media but also enhances the interaction of polymeric shell and ions which lead to an excellent opportunity for the naked eye detection and removal of these ions. The obtained results in this section indicated that the proposed sensor is a simple, precise, inexpensive, has relatively fast response time, and linear over a broad concentration range.

### UV–Vis

**Figure 12:**
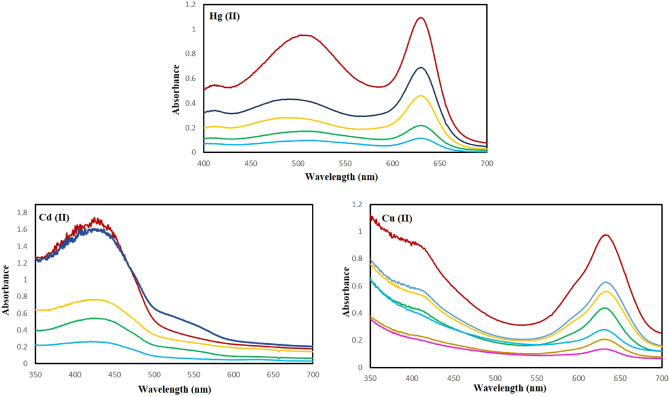
The absorbance spectra observed for the chemosenor with increasing concentrations of Hg (II), Cd (II) and Cu (II) ions at pH values of 8, 9 and 6, respectively, and after equilibrating for 2 min at room temperature.

Apart from the naked-eye sensing, the selective nature of the colorimetric sensor towards the mentioned ions in comparison to other metal ions was confirmed by distinct absorbance bands, in contrast, no similar bands was observed for other metal ions. As can be seen in Fig. 12, by raising the Hg (II), Cd (II), and Cu (II) concentrations, the color intensity of the [metal-dithizone]n + complexes were increased thus confirming the strong interactions and the tight binding of sulfur and nitrogen chelating groups of the DTZ chromogen to metal ions.

### Selectivity

To examine the sensitivity and selectivity along with reliability and high accuracy for ions, several experiments were carried out. The colorimetric detection of the sensor was investigated in the presence of several interference ions including Ca^2+^, Mg^2+^, Zn^2+^, Ni^2+^, Mn^2+^, Fe^3+^, K^+^, and Ag^+^, at varying concentration of Hg (II), Cd (II), Cu (II) and UO_2_ (II) (Table 1). The solutions still exhibited the desired color change; however, Fe^3+^ and Ag^+^ interfered in high concentrations for the detection of ions. The obtained results indicate that the colorimetric sensor has excellent selectivity toward Hg (II), Cd (II), Cu (II), and UO_2_ (II) over interference ions under the optimal conditions.

**Table 1:** The selectivity investigated of chemosensor on the recognition of 1 ppm Hg (II), Cd (II), Cu (II) and UO_2_ (II) by adding of interfering transition metal ion in optimum condition.

Tolerance limit for various cations (ppm)								
Analytes	Ca^2+^	Mg^2+^	Zn^2+^	Ni^2+^	Mn^2+^	Fe^3+^	K^+^	Ag^+^
Hg^2+^	20	20	5	10	8	5	15	3
Cd^2+^	20	20	5	10	6	5	20	3
Cu^2+^	20	20	4	10	4	2	15	1
UO_2_^2+^	20	20	3	8	4	1	15	1

### Reproducibility

Reproducibility is one of its essential characteristic features in determining the suitability of optical sensors, and this factor was investigated in this work. The reproducibility experiments were examined by synthesis of 4 different time with the same composition and measuring the naked eye and absorbance intensity of each chemosensor under optimum pH and times. The resulting coefficient of variation was found to be ± 1.8%.

### Reversibility

Simple treatment using the 1 mol L^−1^ nitric acid was found to efficiently remove the metal ions. These experiments were carried out several times via the release of metal ions. After several regeneration/reuse cycles (i.e., ≥ 4), although the metal ion-sensing system demonstrated a slight influx on the sensitivity with increased regeneration/reuse cycles of metal-to-dithizone ligand binding, they exhibited good-controlled signaling in the quantification and limit of detection of metal ions.

### Colorimetric assay for the detection of toxic ions in real sample

The design and synthesis of chemosensors are essential when they are effective in practical applications^30–32^. Consequently, to confirm the reliability of this detection assay, sensing toxic ions in drinking, river, sea, and tap water were demonstrated with the colorimetric sensors. The standard and spiked samples of water were checked, and the results confirmed the suitability of the synthesized colorimetric sensor for the rapid detection of ions at trace levels in real samples.

### Adsorption experiments

#### The adsorption capacity

Adsorption study was performed to investigate the adsorption equilibrium under different parameters including the contact time, pH of solution, and initial concentration of analyte to find optimum conditions for the adsorption and removal of Cd ions from aqueous solution.

After finding the optimum conditions, the adsorption capacity of PGNPs was calculated using the following equation: Q = (C_0_–C_F_)V/M Where Q (mg g^−1^), C_0_ (mg L^−1^), C_F_ (mg L^−1^), V (L) and M (g) are adsorption capacity, initial concentration of Cd ions, the final concentration of Cd ions, sample volume and PGNPs mass, respectively.

### Effect of contact time

To assess the effect of contact time on removal of Cd ions, the difference times (5–40 min) were investigated. Maximum adsorption was obtained at 20 min time and there was no significant increase with over time beyond that.

Initially, the excess of accessible active sites are available on the surface of PGNPs, and hence the trapping of Cd ions and adsorption capacity are increasing over time. With the occupation of the active places via the passage of time, further increase on adsorption capacity could not demonstrated.

### Effect of pH

The adsorption of Cd ions onto the PGNPs at different initial pH was examined. The same results were obtained as described earlier for the section of naked eye detection. (The best adsorption behavior was obtained at the pH = 9).

### Effect of initial concentration

The influence of initial concentrations of Cd ions on the adsorption of PGNPs was explored in the range of 10–150 ppm. The increased adsorption capacity was observed in the range of 10–100 ppm and at the higher concentration, it could not show significant effect in adsorption capacity due to occupation of overall functional groups on the surface of the sorbent.

### Adsorption kinetics

**Figure 13:**
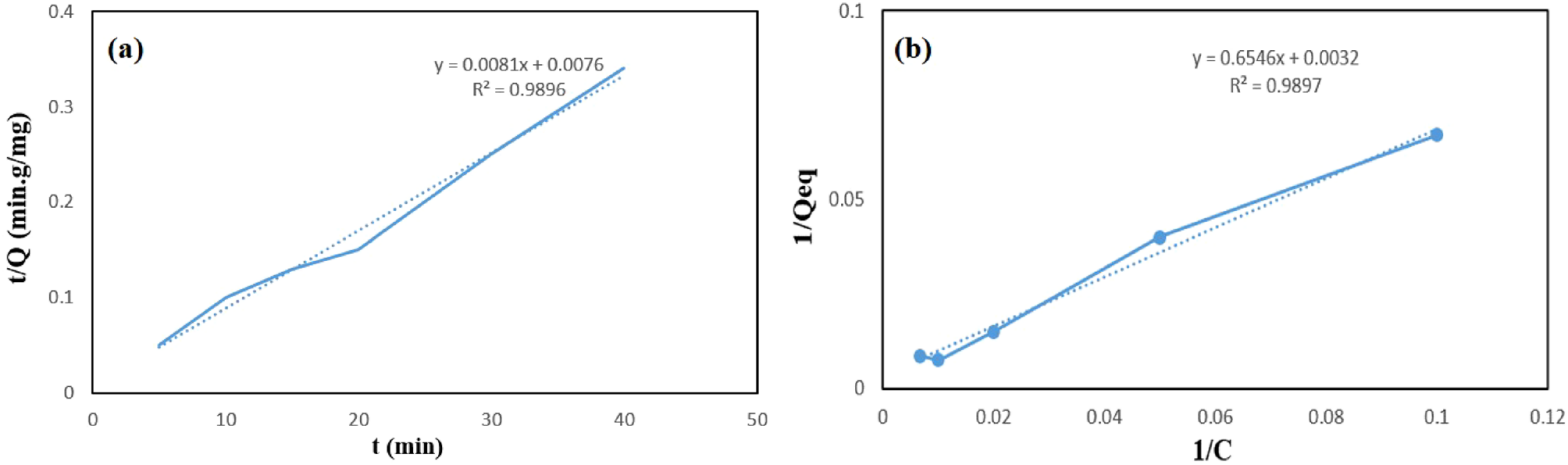
Pseudo second-order plot for PGNPs (**a**) and Langmuir adsorption isotherm plot for PGNPs (**b**).

Kinetics of adsorption process was studied to explain the relationship between time and adsorption capacity, mechanism of adsorption and to determine the equilibrium time. For this purpose, 15 mg of PGNPs was stirred in 100 ppm Cd ion during different time (5, 10, 15, 20, 30, 40 min) intervals. The ensuing data demonstrate an increase in an adsorption amount up to 20 min, as the adsorption process reached the equilibrium and after this time the adsorption capacity (133 mg/g) almost remained constant. Therefore, 20 min was selected as the optimum time. After reaching equilibrium, the available sites of PGNPs are completely occupied, and the additional contact time has no enhancement effect on adsorption. In order to investigate the mechanism of adsorption, the data were fitted on two kinetics models, pseudo-first-order and pseudo-second-order kinetics models which are represented by Eq. (1a) and (1b) respectively^33^:

1a $${\text{ln}}({{\text{Q}}}_{{\text{eq}}}- {{\text{Q}}}_{{\text{t}}})={{\text{lnQ}}}_{{\text{eq}}}-{{\text{K}}}_{1}{\text{t}}$$

1b $$\frac{t}{{{\text{Q}}}_{{\text{t}}}}=\frac{1}{{{\text{K}}}_{2}{{\text{Q}}}_{{\text{eq}}}^{2}}+\frac{{\text{t}}}{{{\text{Q}}}_{{\text{eq}}}},$$

where Q_eq_ (mg g^−1^), Q_t_ (mg g^−1^), K_1_ (min^−1^), K_2_ (g mg^−1^ min^−1^) and t (min) are adsorption capacity of sorbent in equilibrium time, adsorption capacity measured at time (t), pseudo-first-order kinetics rate constants, pseudo-second-order kinetics rate constants and adsorption time, respectively^34^. According to the data, a higher correlation coefficient (R^2^ = 0.98) was obtained for pseudo-second-order kinetics fitted curve, which confirmed the better accordance of this model with the Cd ions adsorption mechanism (Fig. 13a and Table. 2).

**Table 2:** Pseudo second-order constants for PGNPs.

Experimental Q (mg g^−1^)	Pseudo-second-order model		
	K_2_ (g mg^−1^ min^−1^)	Q_eq_ (mg g^−1^)	R^2^
133	0.0071	131	0.98

### Isotherms experiments

Isotherms experiments were applied to investigate the effect of initial concentration of Cd ions on adsorption capacity of sorbent. The PGNPs (15 mg) was stirred with the different concentration of Cd ions in the range of 10 to 150 ppm; measurements demonstrated the increase in adsorption capacity of PGNPs where the Cd ion concentration increased in solution. Adsorption amount increased up to 100 ppm of ions and more concentrations had no increasing effect on adsorption capacity of sorbent. Therefore 100 ppm was considered as the optimum concentration of Cd ions in adsorption process.

To characterize the adsorption mechanism, two isotherm models—Langmuir and Freundlich—were used. Langmuir and Freundlich models are simply represented by the following equations^35,36^:

2a $$1/{Q}_{eq}=1/{Q}_{m}{K}_{l}.1/{C}_{eq}+1/{Q}_{m},$$

2b $${\text{log}}{Q}_{eq}=1/{n}_{f}{\text{log}}{C}_{eq}+{\text{log}}{K}_{f},$$

where Q_eq_, Q_m_, K_l_, C_eq_, K_f_ and n_f_ are the adsorption capacity at equilibrium, maximum adsorption capacity, Langmuir constant, Cd ions concentration at equilibrium and Freundlich adsorption constants, respectively. Langmuir model explains a monolayer adsorption on homogeneous identical sites of surface whereas Freundlich model pertains a multi-layer adsorption process on a heterogeneous surface by an empirical equation. The Langmuir model, which has a higher R^2^ (R^2^ = 0.98), is suggested to be a more effective model to represent the sorbent adsorption data based on the R^2^ results for both models. Hence, Cd ions adsorption is considered as a monolayer adsorption on homogeneous identical sites of PGNPs (Fig. 13b).

### Comparison of colorimetric sensor with existing reports

Herein, we have compared our colorimetric sensor with other documented sensors that have been described; comparison is summarized in Table 3^37–40^. This introduced chemosensor indicated ultrasensitive nature and dual functional process for rapid and selective detection of multi-ions in aqueous media without the need for the expensive instruments.

**Table 3:** Comparison of proposed colorimetric sensor with the other literatures.

Synthesized sensor	Response time (min)	Color change	LOD Hg^2+^	LOD Cu^2+^	LOD Cd^2+^	Ref
1	5	Colorless to pink	1.71 μM	–	–	37
2	15	Blue to mauve	450 ppb	–	–	38
3	–	Colorless to yellow	–	170	–	39
4	10	Red to blue	–		5 μM	40
5	5	Gray to violet	10 ppb	0.5 ppb	1 ppb	This work
	5	Gray to orange				This work
	5	Gray to green				This work

## Conclusion

In line with WHO’s strategic goal of rapidly identifying toxic ions in solutions, we have introduced a novel colorimetric sensor as an ultrasensitive, selective, dual functional colorimetric, and quantitative indicator that functioned well in the presence of interferences ions. The detection limits for Hg (II), Cd (II), Cu (II), and UO_2_ (II) were calculated to be 10, 1, 0.5, and 1 ppb, respectively, which were below the safety limit in tap water and were superior to that of most of the reported values in the literature. Since the poly(AM-MBA-DTZ)-grafted TiO_2_ NPs is highly dispersed in aqueous solutions, the metal-complexation and color change of the chemosensor could be efficiently observed. The color of the chemosensor in the presence of Hg (II), Cd (II), Cu (II), and UO_2_ (II) shifted from gray to violet, orange, green and yellow, respectively. Performance characteristics of the evaluated chemosensor identified it as an excellent sensor with capability of multi-target ions recognition over a wide linear dynamic range, being reversible and stable with short response time.

## Data Availability

All data generated or analyzed during this study are included in this published article.
